# Supplementary material for: A real-world multicenter cross-sectional observational study to assess the clinical profile of peripheral neuropathy in patients with diabetes
Source: PLoS One. 2025 Apr 15;20(4):e0312085. doi: 10.1371/journal.pone.0312085 (PMC11999154; doi:10.1371/journal.pone.0312085)
Supplement: S1 File — (DOCX) [file pone.0312085.s001.docx]

**Details of Diabetic Foot scanner Tool**

The Kit consisted of a concave foot examination mirror to be placed on the floor or surface where the patient can comfortably rest their feet.

The concave shape will magnify and focus the reflection, allowing the doctor to remain seated and observe details of the foot more clearly.

Design Requirements

1. Concave Mirror Design: Shape: A concave (curved inward) mirror, which will magnify the reflection of the feet, allowing the doctor to see a more detailed view of specific areas.

Material: Use durable, high-quality, anti-shatter glass or acrylic with reflective coating for clarity and safety.

Size: The mirror should be large enough to reflect both feet at once, allowing the doctor to examine them simultaneously.

Dimensions: 10–14 inches in diameter with a moderate concave depth for magnification.

2. Base and Frame:

Surface Platform: The mirror was integrated into a platform where the patient can place their feet. The platform should be slightly raised, ensuring the patient's feet are centered on the concave mirror.

Tilt Adjustment: Allow for some tilt adjustment of the mirror to change the viewing angle without moving the patient's feet. This will help the doctor get a better view from different angles.

Frame Material: The frame and platform should be lightweight but durable, made from materials like aluminum or ABS plastic, ensuring stability when the patient rests their feet.

3. Magnification Focus: The concave mirror naturally magnifies the reflection, allowing the doctor to focus on specific parts of the foot (such as the heel, arch, or toes) with greater detail. The curvature should be moderate, not too deep, so the magnification is useful but not distorted.

4. Portability and Stability:

Portability: The device was lightweight and easy to move between examination rooms, allowing for flexibility in clinic use.

Non-Slip Base: The bottom of the platform should have non-slip rubber pads to ensure the platform stays securely in place while the patient rests their feet on it.

5. Lighting: To enhance visibility, especially for inspecting foot ulcers, skin conditions, or deformities, incorporate LED lighting around the perimeter of the mirror. These lights can illuminate the feet while minimizing shadows. Optionally, include a dimming feature to control lighting intensity based on the clinic's ambient light.

6. Hygiene and Sanitation:

Sanitary Foot Pad: Place a replaceable or washable foot pad on the platform to ensure that the patient’s feet remain in a clean environment. The pad should be made from a material that can be sanitized between uses.

Antimicrobial Coating: Consider applying an antimicrobial coating on the surface of the platform to reduce the risk of infection.

Functionality and Use Case • Patient Position: The patient will place their feet on the platform, with the concave mirror reflecting the sole of the foot upward toward the doctor. • Doctor’s Position: The doctor remains seated, using the concave mirror's magnified reflection to observe the soles of the feet. The magnification allows a more detailed inspection of areas prone to foot ulcers, calluses, or other skin conditions. • Enhanced View: The magnified reflection provided by the concave mirror helps the doctor clearly see small cracks, lesions, or abnormalities, improving diagnostic accuracy for conditions such as diabetic foot ulcers.

Benefits • Enhanced Detail: The concave shape naturally magnifies the foot’s reflection, providing an in-depth view of hard-to-see areas like the heel, arch, and toes without the need for additional equipment.

• Comfort: Both the doctor and the patient remain comfortable throughout the examination, with minimal physical movement.

• Convenient Diagnosis: The doctor can easily assess foot conditions like ulcers, infections, or abnormalities with greater precision, making it ideal for diabetic foot care and other conditions.

Design Elements • Platform Dimensions: 14 x 10 inches to fit comfortably under both feet. • Concave Mirror Radius: A 10-inch diameter mirror with a moderate concave depth for proper magnification without distortion. • Weight: The platform weighed less than 1 kg for ease of movement. • Adjustability: A tilt adjustment lever or dial for fine-tuning the angle of the reflection.
