# Supplementary material for: A real-world multicenter cross-sectional observational study to assess the clinical profile of peripheral neuropathy in patients with diabetes
Source: PLoS One. 2025 Apr 15;20(4):e0312085. doi: 10.1371/journal.pone.0312085 (PMC11999154; doi:10.1371/journal.pone.0312085)
Supplement: Attachment — Submitted filename: Rebutal letter_AR_WJV.docx [file pone.0312085.s003.docx]

­**Response to the reviewers**

**We sincerely thank the editor and reviewers for spending their valuable time to review our manuscript and for providing their comments, which helped to improve the quality of the manuscript.**

**Point by point clarification/corrections to the concerns raised by the editor and reviewers are summarized. The changes in the revised manuscript are highlighted Red**

Overall this is a large and important contribution to the literature in relation to identifying DPN and foot problems in India.

1. The background should not be: Background: Increasing Type 2 Diabetes Mellitus (T2DM) in Asian-Indian populations highlights a gap in understanding diabetic peripheral neuropathy (DPN) and related foot deformities. Rather it should be: There are limited studies on the prevalence of diabetic peripheral neuropathy (DPN) and related foot deformities in patients with T2DM from India.

We thank the reviewer for the suggestion and have changed accordingly.

2. In the methods you need to specify the study setting i.e. primary care, secondary care or tertiary care hospital?

We have added the following sentence: “Data collection for the study was conducted between October 8, 2023, and November 20, 2023, with patients coming from primary, secondary, and tertiary care settings.”

3. How many centers were involved and which part of India were they from?

In the Material and Methods section we have added the following:

Data collection for the study was conducted between October 8, 2023, and November 20, 2023, with patients coming from primary, secondary, and tertiary care settings across 543 centers in India, covering the following states: Jammu, Kashmir, Punjab, Rajasthan, Delhi, Bihar, Uttar Pradesh, Madhya Pradesh, Odisha, West Bengal, Assam, Andhra Pradesh, Tamil Nadu, Karnataka, Kerala, Maharashtra, and Gujarat.

4. When assessing neuropathic symptoms was any particular validated questionnaire such as DN4 used? The diagnosis of neuropathy in this study was based solely on the physician’s judgment. While this is common practice in India, it may have introduced some variability. A more standardized approach, such as using a validated questionnaire like the DN4 @bouhassira_comparison_2005 for diagnosing neuropathy, would have been better to ensure consistency.
5. Details on the ‘Diabetic foot Scanner’ are required.

**We have provided a link to [www.eris.co.in/diabetic-care](http://www.eris.co.in/diabetic-care) , which includes all relevant information about the device.**

6. How was ‘neuropathy’ diagnosed?

Neuropathy was diagnosed based on clinical judgement of practicing clinicians. We recognize that this is a limitation of the study and have mentioned this in the limitation section. “The diagnosis of neuropathy in this study was based solely on the physician’s judgment. While this is common practice in India, it may have introduced some variability. A more standardized approach, such as using a validated questionnaire like the DN4 @bouhassira_comparison_2005 for diagnosing neuropathy, would have been better to ensure consistency”

7. The prevalence of diabetic retinopathy (12.9%) compared to nephropathy (27.2%) seems very low. How were these defined, from the medical notes or history? In the discussion, we included a section on “Diabetic Peripheral Neuropathy and Comorbidities” to clarify that the results are based on patients' medical histories. Additionally, we explored potential reasons for the underreporting of retinopathy compared to nephropathy.
8. The prevalence of infection is very high (19.7%). Therefore were these patients at a specialist foot clinic?

Most of the centers are diabetes clinics, and patients were physically examined especially foot examination. Specialized diabetic foot clinic is not the usual norm in this part of the world and diabetes clinic provide foot care services as well which may not be called “specialized foot clinic” In the discussion section we added under the subheading: “Prevalence of Foot deformities” an explanation for the high number of patients with infection.

9. The discussion needs to be a discussion where the data from the current study has to be compared to previous papers from India or elsewhere. Currently it is simply a repeat of the results without any context. Thus, if 35.4% had burning symptoms, how does this compare to other studies?

We thank the reviewer for this critical and valuable comment. We have checked for redundancy. Therefore we have rewritten major parts of the discussion section. We have mentioned the most important results included prevalence values and explained how they compare to previous studies.

10. In the discussion you do not need to refer to the P value in previous studies.

We agree and have removed the p-values accordingly

11. With regard to dyslipidemia, the discussion should focus on its relationship to DPN and DFU, not with CAD.

We agree with the reviewer’s opinion and have removed the specific part of CAD relating to dyslipidemia. We only focused on the relationship of dyslipidemia with Foot health/DFU.

12. Dry skin is not a good indicator of PAD, but represents sudomotor dysfunction.

We agree and have edited the sentence: “This suggests it may be linked to reduced blood flow in PAD, although the association is not conclusive.”

We hope that the revised manuscript may be considered suitable for acceptance.

Sincerely,

Ashu Rastogi
